# Gamified Text Messaging Contingent on Device-Measured Steps: Randomized Feasibility Study of a Physical Activity Intervention for Cancer Survivors

**DOI:** 10.2196/18364

**Published:** 2020-11-24

**Authors:** Michael C Robertson, Elizabeth J Lyons, Yue Liao, Miranda L Baum, Karen M Basen-Engquist

**Affiliations:** 1 Department of Behavioral Science University of Texas MD Anderson Cancer Center Houston, TX United States; 2 Health Promotion & Behavioral Sciences University of Texas School of Public Health Houston, TX United States; 3 Department of Nutrition and Metabolism School of Health Professions The University of Texas Medical Branch at Galveston Galveston, TX United States; 4 Department of Kinesiology College of Nursing and Health Innovation University of Texas at Arlington Arlington, TX United States

**Keywords:** cancer survivors, physical activity, motivation, self-control, mobile health, mobile phone, technology

## Abstract

**Background:**

Physical activity can confer diverse benefits on cancer survivors. Unfortunately, many cancer survivors are not sufficiently active. The efficacy of physical activity interventions for this population may be increased by grounding them in Self-Determination Theory (SDT). Combining game design elements with wearable technologies may be a useful and scalable approach to targeting SDT constructs to promote cancer survivors’ physical activity.

**Objective:**

The primary aim of this study is to evaluate the feasibility and acceptability of *Steps2Health*, a physical activity intervention for cancer survivors. It also aims to investigate the effects of the intervention on motivation, physical activity, and step count.

**Methods:**

We randomized 78 insufficiently active cancer survivors to an experimental or comparison group. All participants received a physical activity tracker. The experimental group participants also received a set sequence of multimedia messaging service messages that were triggered in real time by meeting predetermined cumulative step count totals. Messages presented information about a virtual journey and included photographs and vivid descriptions of locations to increase autonomous motivation. Additional messages targeted perceptions of *relatedness* (eg, role modeling) and *competence* (eg, facilitating mastery experiences). We administered pre- and postintervention surveys and conducted 15 individual interviews to evaluate the intervention. We performed directed content analysis of qualitative data and conducted mixed effects linear modeling to investigate participants’ changes in motivation, self-reported physical activity, and device-measured step counts.

**Results:**

There was minimal loss to follow-up (3/78, 4%), the device wear rate was high (2548/3044, 83.71% of days), and technical problems with messaging based on real-time step counts were limited. Our qualitative data analysis revealed 3 overarching themes: *accessibility*, *autonomous motivation*, and *relatedness*. Participants successfully navigated the technological aspects and game design elements of the intervention. Participants found messages targeting *autonomous motivation* and *competence or self-efficacy* to be enjoyable and compelling, but one feasibility criterion for participant engagement (response rate to text messages) was not met. Messages targeting *relatedness* were less highly rated than the messages targeting *autonomous motivation* and *competence or self-efficacy*. During the intervention, both groups increased their motivation for physical activity (*B*=0.16; 95% CI 0.01 to 0.30; *P*=.04; *d*=0.49), and assignment to the experimental group was associated with increased self-reported leisure activity score (*B*=10.78; 95% CI 3.54 to 18.02; *P*=.005; *d*=0.64). The experimental group had greater increases in daily step counts over time (*B*=322.08; 95% CI 54.01 to 590.15; *P*=.02; *d*=0.28).

**Conclusions:**

This study supports the feasibility of using real-time game design elements to target SDT constructs and increase cancer survivors’ physical activity. Overall, our findings support the acceptability of the *Steps2Health* intervention, but fostering active participant engagement and targeting *relatedness* may present additional challenges. *Steps2Health* may help cancer survivors increase their physical activity levels.

## Introduction

### Background

Physical activity is generally safe and a health protective factor for cancer survivors. It is associated with a lower risk of all-cause mortality in this population, and for survivors of some types of cancer, it is associated with lower risks of recurrence and cancer-related mortality [1]. Increasing moderate-to-vigorous physical activity may ameliorate symptoms that cancer survivors commonly report, including fatigue, pain, anxiety, decreased physical functioning, and cancer-related cognitive impairment [2]. Unfortunately, the majority of cancer survivors do not meet the nationally recommended aerobic physical activity guidelines for adults [3], which call for engaging in 150 min of moderate-intensity aerobic physical activity or 75 min of vigorous-intensity aerobic physical activity (or some equivalent combination of both) per week. An analysis of data from adult cancer survivors responding to the 2014 National Health Interview Survey indicated that under 45% of adult survivors aged 45-64 years met the guidelines for aerobic physical activity, and this percentage was approximately 35% in survivors 65 years and older [4]. Studies using accelerometers to objectively measure physical activity guideline adherence indicate that this percentage may be between 4% and 13% [5,6]. Cancer survivors may encounter barriers that impede physical activity guideline adherence faced by the general population (eg, competing demands for time) and additional barriers attributable to cancer and its treatment. These can include decreased physical functioning, fatigue, and pain [7,8].

Electronically delivered behavioral interventions have been shown to be effective in promoting physical activity in cancer survivors [9]. Such interventions readily lend themselves to widespread dissemination and increasingly feature mobile and wearable computing technologies (mobile health [mHealth]) that can provide timely feedback on behavior. Although behavioral interventions incorporating wearable consumer technologies have been used to initiate physical activity among cancer survivors [10], evidence of the long-term effectiveness of these interventions is lacking. Furthermore, most mHealth programs have high discontinuation rates (eg, 25% of users abandon mobile apps after just a single use) [11]. Many mHealth programs are centered on facilitating self-regulatory processes (eg, goal setting, self-monitoring) but are not necessarily designed to affect participants’ *motivation* for physical activity. This is an important distinction, particularly because cancer survivors may face barriers that can diminish their motivation to engage in physical activity (eg, decreased physical functioning, fatigue, pain) [7,8]. Increasing cancer survivors’ motivation for physical activity may facilitate sustained engagement with mHealth programs and improve long-term behavioral adherence to recommended guidelines [12].

Self-Determination Theory (SDT) provides a framework for understanding the role of quality of one’s motivation in long-term adherence to health-related lifestyle behaviors [13]. It posits that motivation can be conceptualized as existing on a continuum that ranges from fully external or controlled motivation to fully internal or autonomous motivation and that increasing the latter tends to yield longer lasting behavioral change than the former [13]. SDT also holds that *autonomous regulation* is determined in large part by the satisfaction of an individual’s core psychological needs (*autonomy, relatedness,* and *competence*) [13]. This theoretical approach can be useful in predicting and influencing physical activity in the general population [14], and targeting SDT constructs may be similarly beneficial for promoting physical activity in cancer survivors [15]. Indeed, SDT is increasingly being used to inform interventions in this population [16,17].

One approach that has been used to effectively target SDT constructs in the general population is gamification, the application of game design elements to nongame contexts [18-20]. Thus, we drew from the games-for-health literature to develop *Steps2Health*, an mHealth intervention that targets SDT constructs to promote cancer survivors’ physical activity. As only few models for systematic planning of gamification interventions exist [21], we adapted the Behaviour Change Wheel model for this purpose [22]. This model typically asks planners to match intervention functions (eg, persuasion) to theoretical constructs (eg, reflective motivation). Our adaptation included playful experiences taken from the Playful Experiences Framework [23] as potential intervention functions. We chose to focus on playful experiences as broad methods of intervention (rather than game mechanics as specific behavior change techniques) to emphasize the autonomy-supportive, playful aspects of games, as has been recommended [21,24]. Specifically, we focused on *the playful experiences of discovery, exploration, and humor to target intrinsic regulation*. In addition, we included techniques from Motivational Interviewing and Acceptance and Commitment Therapy to complement the game messages and target *integrated regulation* [25,26].

### Objectives

The main purpose of this study is to assess the feasibility of *Steps2Health* and the participating cancer survivors’ satisfaction with it. Its secondary objective is to assess the effects of *Steps2Health* on participants’ autonomous regulation, self-reported physical activity, and device-measured physical activity (ie, step counts).

## Methods

### Recruitment

We identified potential participants through various means, including health fairs, conferences, and other local events in South Texas; social media (eg, Facebook, Twitter); and our institutional website and publications. We contacted interested individuals via email and telephone and conducted a formal screening process via telephone. We engaged in a verbal informed consent process with all eligible individuals between September 2018 and February 2019. All research protocols were approved by the University of Texas MD Anderson Cancer Center’s Institutional Review Board (protocol 2018-0239). Participants were adult cancer survivors who had completed primary cancer treatment for at least three months previously, owned a smartphone, and were willing to receive text messages and complete web-based surveys. Eligible participants did not meet the recommended physical activity levels [27] at screening as determined by verbal administration of the modified Godin Leisure-Time Exercise Questionnaire [28].

### Study Design

We conducted a randomized controlled pilot trial. As the intervention duration was contingent on participants’ cumulative step count (ie, participants who registered more daily steps progressed though the intervention more quickly), we assigned participants to the experimental and comparison groups in pairs. We recruited cohorts of 6 participants (to facilitate the logistics of study operations) and randomly assigned pairs within each cohort and group assignment within each pair (ie, for each group of 6 participants, we randomly assigned each participant to 1 of 3 pairs, and for each pair, we randomly assigned one participant to the experimental group and the other to the comparison group). The study staff conducted this restricted randomization procedure using Research Electronic Data Capture (Vanderbilt University). The intervention duration for both participants in each pair was determined by the experimental group participant’s time to reach a predetermined cumulative step count (see the Intervention section). We administered surveys before and after the intervention. For each pair, we continued to record participants’ step counts for 4 weeks after the experimental group participant had completed the intervention. The participants assigned to the control arm completed baseline and follow-up assessments in the same timeframe as their paired counterpart (ie, the Intervention section details the study duration for each pair determined by the activity level of the experimental group participant).

We conducted individual, semistructured interviews with 15 experimental group participants at the end of their study participation. Questions were centered on obtaining feedback on the feasibility and acceptability of the intervention and insights into how it may be improved (Multimedia Appendix 1). We interviewed some participants who completed the intervention most quickly and some who completed it least quickly. We interviewed additional participants at the discretion of the principal investigator (eg, to ensure that we interviewed some men). Each interview lasted for 30 to 60 min, and all interviews were conducted by the first author (MR). The study staff (MB) took detailed notes in all interviews. Immediately after the interview, the first author reviewed and contributed to these notes. We performed qualitative data collection until there was consensus among the research team that the point of data saturation had been reached and the individual interviews were not producing novel content any longer.

### Intervention

All participants engaged in this study remotely. We synchronized wrist-worn Fitbit Alta devices to Fitabase (Small Steps Laps), a web-based data management platform for wearable devices, and then mailed each participant a device. We instructed participants to wear the device during waking hours. We included instructions and links to the Fitbit content detailing how to set up the Fitbit device to synchronize automatically. All participants had access to the Fitbit website and app. In addition, the participants in the experimental group received *Steps2Health* multimedia messaging service (MMS) messages that were designed to target SDT constructs. These messages, which were developed by the research team, presented information about a virtual journey through Japan’s Inland Sea region and were triggered by step counts in real time. Participants’ progress on the 166,000-step (approximately 83 miles) virtual journey was determined by cumulative step counts measured by the Fitbit devices. All experimental group participants received the same series of text messages. The duration of the *Steps2Health* intervention was determined by how long it took participants to register 166,000 steps on their device (ie, if participants were more active, they received the messages more frequently). We chose this distance because we anticipated that it would take most participants about 1 month to complete the journey. We designed the *Steps2Health* intervention to have a variable duration contingent on participant step count to facilitate a sense of autonomy for the user and verisimilitude for the gamified intervention’s premise of undertaking an actual journey in real time. There is only one journey featured in this pilot study, but ultimately, *Steps2Health* may feature a variety of journey options that users can engage in sequentially for a more continuous experience.

The *Steps2Health* intervention included 54 total messaging blocks designed to target *autonomous regulation*, *autonomy*, *relatedness*, and *competence* (Multimedia Appendix 2). Messages were informed by insight from previous qualitative research in cancer survivors and developed in a consensus-building process among the research team, which has expertise in health behavior change theory and physical activity promotion for cancer survivors [29]. All messaging blocks contained text, and some additionally contained images or hyperlinks (Table 1). Each block included 1 to 3 messages. Messages targeting *autonomous regulation* presented photographs and vivid descriptions of destinations along a geographically accurate virtual tour of the region. These messages presented playful experiences of exploration, discovery, and humor and encouraged participants to identify value-based life goals linked to physical activity (Table 1). Some messages contained hyperlinks to various resources for healthy living (eg, videos demonstrating muscle strengthening exercises, healthy recipes local to the current virtual location, stress-reduction techniques, etc). We designed the *Steps2Health* intervention to most heavily target this construct (devoting 21 of 54 messaging blocks to it), given its emphasis on SDT and its central role in the gamification elements of the intervention. Participants were also given the choice to participate in additional mini journeys (13 messaging blocks). These optional messages were similar to those targeting *autonomous regulation*. To target the SDT construct of *relatedness,* we included messages in 10 messaging blocks from Ruby, an ovarian cancer survivor (this was a fictitious character, and participants were made aware of this). This character was written to be a positive role model. Her messages provided encouragement and prompted participants to reflect on questions that were derived from motivational interviewing principles to elicit positive *change talk* [26]. Finally, 10 messaging blocks included messages adapted from previous studies to increase participants’ *competence or self-efficacy* for increasing physical activity.

**Table 1:** Example messages targeting Self-Determination Theory constructs.

Step count	Example messages	Image	Construct targeted
1	Welcome to Steps2Health! Please save this number in your phone as Steps2Health, and be sure that your Fitbit is up set to sync automatically. Bridges serve as major checkpoints for this 83 mile island-hopping trek through beautiful Japanese islands. Keep your step count high to maximize your progress!	Starting message image 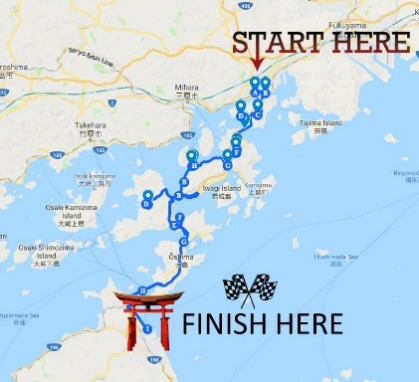	Autonomous motivation
8000	RUBY: Hello! My name is Ruby. I am an ovarian cancer survivor and have already completed this journey. I wanted to get strong to keep up with my grandson. Is there a goal you’d like to work toward? Would you share it with me in a text? If not, just text 0.	Relatedness example message image 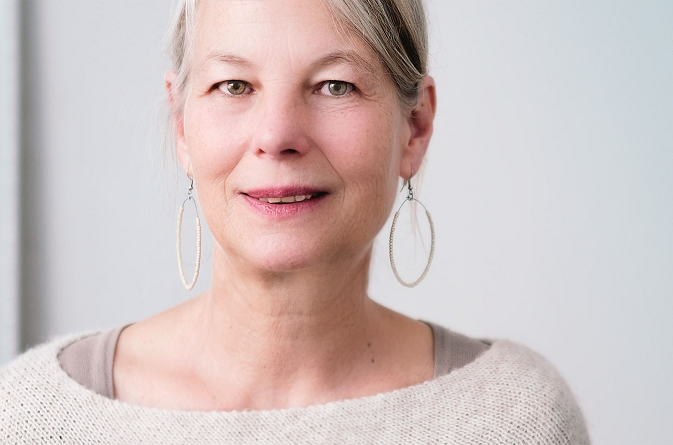	Relatedness
45,000	Would you like to take a quick trip to Onomichi, the “Town of Hills and Cats” today? You'll get some extra photos of high points of Onomichi. Reply YES or NO	N/A^a^	Autonomy or autonomous motivation
57,300	You have made it to the beautiful Kosanji temple. It was built in 1936 by a wealthy industrialist in honor of his mother! It is written in a famous haiku: The mothers of the world are as the Goddess of Mercy.	Autonomous motivation example message image 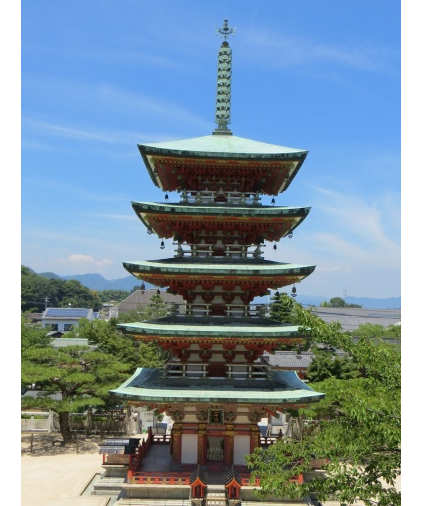	Autonomous motivation
84,000	HEALTH TIP: Living through cancer can be stressful, but you can manage the stress. Even a 10-minute time-out can help by taking time to move and breathe.	N/A	Competence or self-efficacy

^a^N/A: not applicable.

We used Fitabase to gather participants’ data in real time from the Fitbit server. We worked with Mosio to build a platform to send intervention text messages on the basis of participants’ real-time step counts. This platform queried Fitabase twice an hour to determine participants’ cumulative step counts. We ensured that participants enabled their devices to frequently and automatically upload current data to the Fitbit server (ie, synchronize with the server). The text messaging platform sent automatic text message reminders for participants to synchronize their devices if they had not done so in 2 days or more, and study staff sent email reminders after 5 days.

### Outcomes

#### Overview

For the primary aim of this study, we evaluated intervention feasibility and acceptability via quantitative and qualitative methods. For the secondary aim, we evaluated changes in autonomous regulation, self-reported moderate-to-vigorous physical activity, and device-measured step count over the course of the intervention period. We administered web-based surveys to each participant at baseline and after the experimental group participants completed the *Steps2Health* intervention. The surveys contained items pertaining to participants’ characteristics, the feasibility of the intervention, and participants’ autonomous motivation and physical activity.

#### Intervention Feasibility

We evaluated feasibility by assessing whether (1) more than 75% (59/78) of all participants wore their Fitbit at least 5 days a week for at least 75% of the study period (*valid wear*); (2) less than 30% (23/78) of all participants reported technical difficulties with the Fitbit or receipt of the text messages (*technical difficulties*); (3) the experimental group participants responded to at least 80.1% (157/196) of the messages requiring a response (*participant engagement*); and (4) at least 75% (30/39) of the experimental group participants reported that they would recommend the program to friends or family (*participant satisfaction*). These criteria were derived from metrics for assessing the feasibility of consumer-based wearable physical activity trackers used in other digital behavior change studies [30] and our intervention’s theoretical orientation.

#### Autonomous Regulation

We administered the Behavioral Regulation for Exercise Questionnaire-3 (BREQ-3) to all participants before and after the intervention. BREQ-3 has acceptable internal consistency [31]. As SDT posits that *autonomous regulation* is composed of identified regulation, integrated regulation, and intrinsic regulation, we averaged these subscale scores to represent this construct [13].

#### Physical Activity

We administered the Godin Leisure-Time Exercise Questionnaire and calculated the Leisure Score Index to measure participants’ moderate-to-vigorous–intensity physical activity [28]. This survey has minimal participant burden, good test-retest reliability (reliability coefficient=0.81), and convergent validity with VO_2_ max (maximum rate of oxygen consumption during intense exercise) [28,32]. In addition, we evaluated participants’ daily Fitbit step count trends. In consideration of the insufficiently active sample recruited in this study and to maximize the use of available data, we used 2 variables to define nonvalid wear days. We defined a nonvalid wear day as a day during which participants (1) did not wear the device for at least 10 hours (out of the full 24-hour day) according to minute-level device data and (2) registered fewer than 1500 steps according to day-level data [33-35]. Minute-level nonwear was defined as periods of 90 consecutive minutes of 0 steps with a 2-min nonzero tolerance, consistent with commonly used accelerometer protocols [36]. We used a combination of day- and minute-level data because some research indicates that the use of accelerometer protocols for minute-level data may tend to overestimate nonwear in Fitbit data [37], and Fitbit devices are programmed to automatically delete minute-level data when their batteries run low or if the devices are not regularly synchronized. Thus, we supplemented this decision rule with the 1500 steps day-level threshold used in other studies [34,35].

### Data Analysis

Two analysts (MR and MB) conducted directed content analysis [38] of the field notes from the individual interviews. We created and assigned inductive and deductive codes to discrete points made by each participant. We organized these codes in an iterative process to gain insight into the perceived feasibility and acceptability of the intervention and means by which the intervention might be improved.

We calculated descriptive statistics for participant characteristics and feasibility items and performed Pearson chi-square tests to evaluate differences in descriptive characteristics between groups. We used linear mixed models to assess between-group differences in pre- to postintervention changes in *autonomous regulation* (as assessed using the BREQ-3) and physical activity (as assessed using the Godin Leisure-Time Exercise Questionnaire Leisure Score Index). These models included terms for group-by-time interactions with random coefficients for participants nested within pairs. We used a linear growth model with random intercepts and slopes to assess between-group differences in changes in Fitbit-measured step counts during the intervention [39]. As the intervention duration varied among pairs, we modeled each participant’s average daily step count as a function of quartiles of the intervention period. We specified an autoregressive covariance structure because of the time series nature of the data and adjusted for the number of days of valid Fitbit wear. We used the maximum likelihood estimation for all linear mixed models. We performed a likelihood ratio test to determine if it was necessary to specify the third level in the linear growth model (pairs subsuming individuals). The results of this test did not show a statistically significant difference in model fit between the 3-level model and a 2-level model (*P*=.90); furthermore, the conclusions to be drawn from the results of the competing models were not substantively different. Accordingly, we present results from the more parsimonious 2-level model with observations nested in individuals. We created plots recommended by Bolger and Laurenceau [40] to analyze and present the linear growth model results. We supplemented the mixed model findings with Cohen *d* effect size calculations. We set the nominal α value to .05 for all analyses, which we performed in R version 3.6.1.

## Results

### Participants

**Figure 1:**
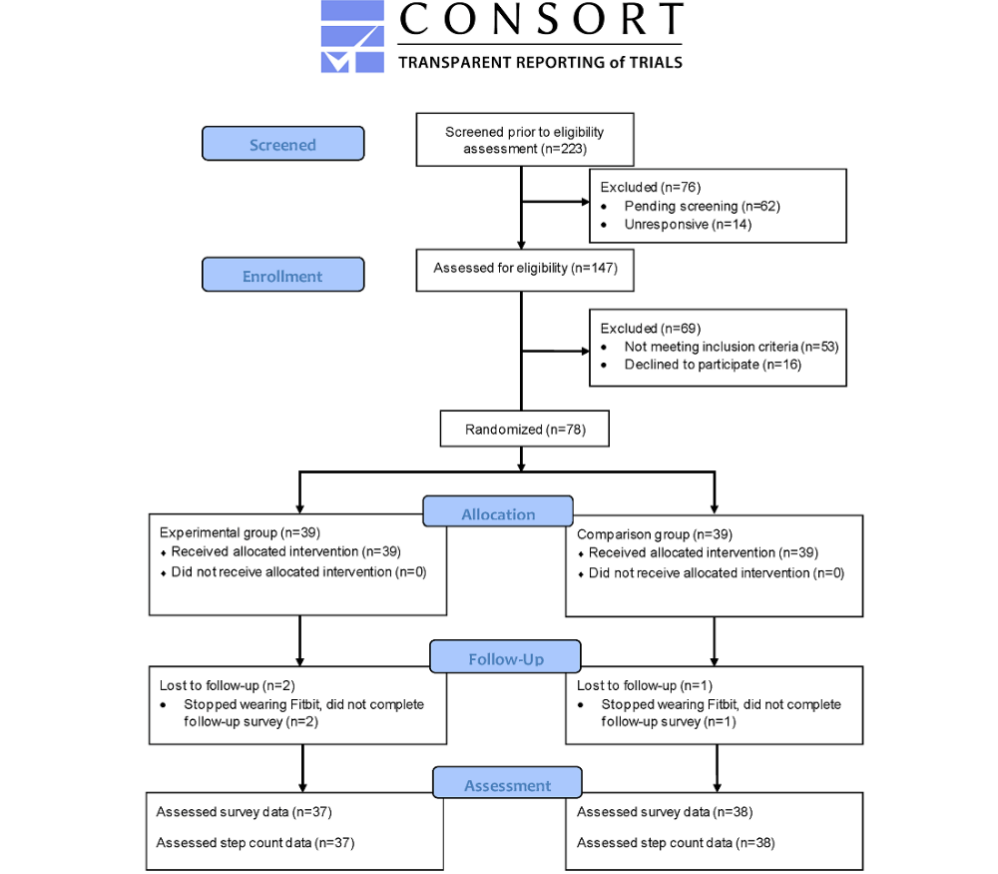
The CONSORT (Consolidated Standards of Reporting Trials) diagram for Steps2Health recruitment, retention, and analysis.

We randomized 78 participants to either the experimental group or comparison group. Of these 78 participants, 3 (4%) were lost to follow-up and 75 (96%) completed the baseline and follow-up surveys (Figure 1). The study sample was mostly female (71/78, 91%) and relatively well educated (Table 2). The participants’ mean age was 55.1 (SD 13.5) years. Most participants were breast cancer survivors (45/78, 58%) and overweight (38/75, 50%) or obese (19/75, 25%). The overall mean time since cancer diagnosis was 9.4 (SD 7.3) years. All participants in the experimental group who completed the preintervention survey also completed the 166,000-step journey.

**Table 2:** Participant characteristics (N=78).

Characteristics		Experimental group, n (%)	Comparison group, n (%)	*P* value^a^
**Age (years)**				.89
	18-34	2 (5)	2 (5)	
	35-49	10 (26)	14 (36)	
	50-64	13 (33)	12 (31)	
	65-74	13 (33)	10 (26)	
	≥75	1 (3)	1 (3)	
**Education level**				.16
	<High school	0 (0)	1 (3)	
	High school diploma or general educational development	5 (13)	2 (5)	
	Some college	11 (28)	12 (31)	
	Bachelor’s degree	14 (36)	10 (26)	
	Graduate school degree	9 (23)	14 (36)	
**Employment status**				.98
	Employed full time	15 (34)	18 (42)	
	Employed part time	8 (18)	6 (14)	
	Not employed for pay, not seeking paid employment	2 (5)	1 (2)	
	Not employed for pay, but seeking paid employment	2 (5)	1 (2)	
	Retired	8 (18)	7 (16)	
	Homemaker	6 (14)	7 (16)	
	Student	1 (2)	1 (2)	
	Volunteer	2 (5)	2 (5)	
**Gender**				.99
	Female	36 (92)	35 (90)	
	Male	3 (8)	4 (10)	
**Marital status**				.62
	Single	9 (23)	5 (13)	
	Married	23 (59)	24 (62)	
	Divorced	6 (15)	9 (23)	
	Widowed	1 (3)	1 (3)	
**Race**				.50
	American Indian or Alaska native	1 (3)	0 (0)	
	Asian	0 (0)	0 (0)	
	Black or African American	8 (21)	6 (15)	
	White	30 (77)	32 (82)	
	Other	0 (0)	1 (3)	
**Ethnicity^b^**				.57
	Hispanic	8 (21)	7 (18)	
	Non-Hispanic	30 (79)	32 (82)	
**Cancer diagnosis**				.46
	Breast	20 (51)	25 (64)	
	Ovarian	4 (10)	3 (7)	
	Colorectal	1 (3)	3 (7)	
	Endometrial	6 (15)	2 (5)	
	Prostate	2 (5)	2 (5)	
	Urinary tract	0 (0)	1 (2)	
	Renal or pelvic	1 (3)	0 (0)	
	Brain	0 (0)	1 (2)	
	Other	6 (15)	6 (15)	
**Body mass index status^c^**				.51
	Normal	11 (30)	7 (18)	
	Overweight	17 (46)	21 (55)	
	Obese	9 (24)	10 (26)	

^a^Pearson chi-square test.

^b^Over 98% (77/78) responded to this item.

^c^Over 96% (75/78) responded to required items.

### Device Wear

The median number of days to complete the journey for experimental group participants was 30; this ranged from 15 to 128 days (IQR 23-51). The overall day-level percentage of valid Fitbit device wear was 83.71% (2548/3044). At the week level, 79% (59/75) of participants wore their Fitbit at least 5 days a week for at least 75% of the overall study period. This exceeded the a priori target of >75%, supporting the feasibility of intervention. During the intervention period, the average percentage of nonvalid wear days was 13.5% for the experimental group and 8.8% for the comparison group. During the follow-up period, these percentages were 27.5% and 20.1%, respectively.

### Intervention Feasibility

We conducted in-depth interviews with 15 participants in the experimental group (12 females and 3 males). Qualitative data analysis revealed 3 overarching themes: *accessibility*, *autonomous motivation*, and *relatedness*.

#### Theme 1: Accessibility

##### Limited Technical Difficulties

Findings from qualitative and quantitative data analyses indicated that participating cancer survivors perceived the *Steps2Health* intervention to be accessible. Regarding the intervention’s technological aspects, participants perceived the intervention to be straightforward and practicable. One participant stated:

Not a single issue. Worked straight up no problem…it was remarkable to me that there were no issues at all.

P60

**Figure 2:**
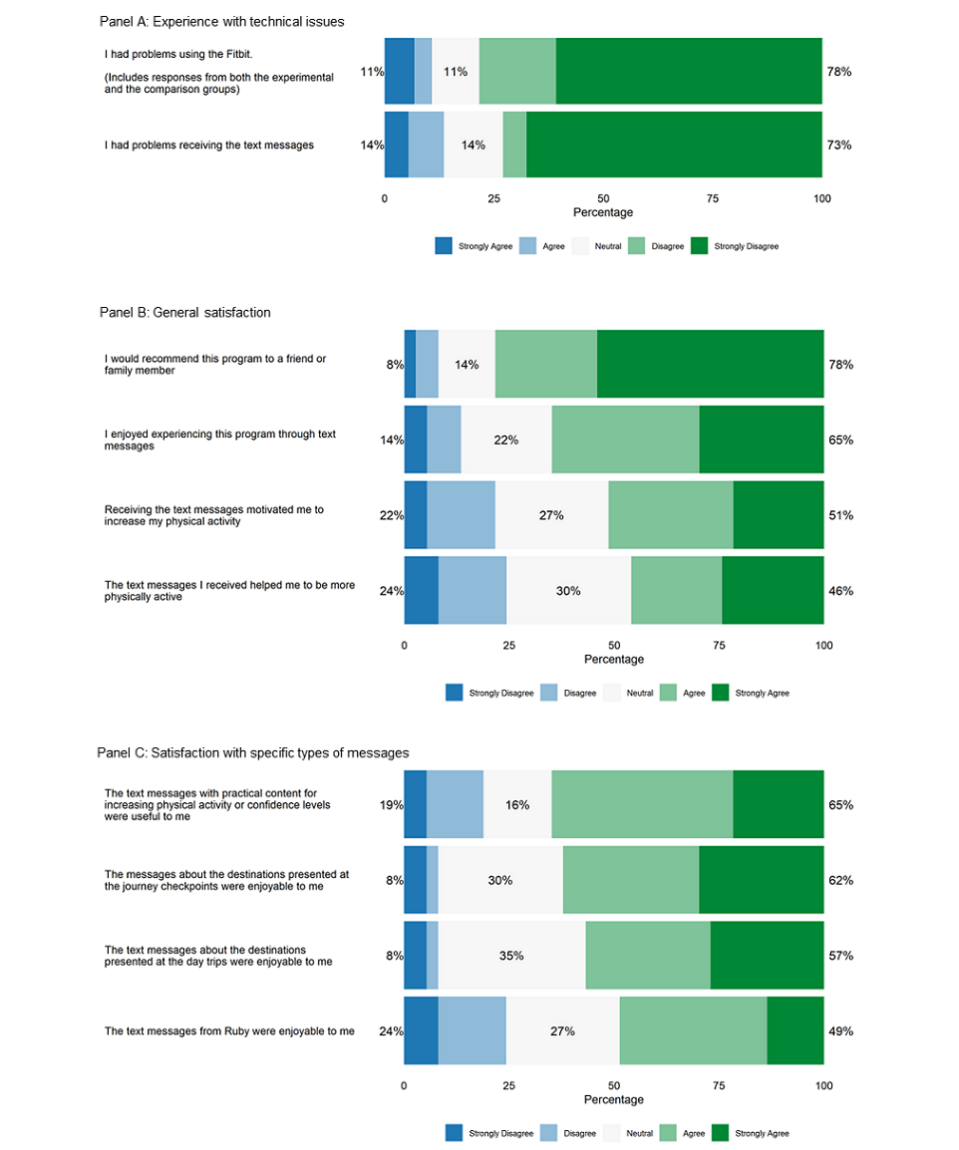
Participant satisfaction with the Steps2Health intervention.

This sentiment is generally supported by participants’ survey data. Quantitative data revealed that only 11% (8/74) of participants indicated that they had problems with using the Fitbit device, and only 14% (10/74) indicated that they had problems receiving the text messages (Figure 2). These numbers were below the a priori target of <30%, supporting intervention feasibility. Technical difficulties included 4 instances of faulty hardware; in each case, the Fitbit customer support team ultimately replaced the device. One early technical issue was that the MMS messages requiring a response were repeated. This was remedied for the third (6 persons) cohort of participants. There was one instance when all intervention messages were sent at once to a participant. With the participant’s permission, we were able to get this person resituated on the journey.

During interviews, several participants suggested providing a more thorough orientation process that would more fully reveal exactly how the text messaging platform worked. In general, participants who offered this suggestion reported that if they did not synchronize their devices frequently, they would receive a burst of several text message blocks in quick succession when they next synchronized their devices. Participants who experienced this said that a more thorough understanding of the intervention messaging mechanism would help them to better navigate these instances and encourage them to synchronize their devices more regularly.

##### Celebratory Orientation

Participants appreciated the *Steps2Health* design that celebrated participants meeting their cumulative physical activity accomplishments and rewarded them with fun and interesting content. This orientation to physical activity promotion made the *Steps2Health* intervention feel like an accessible program that accommodates participants’ fitness levels. One participant said:

I’m not real big on exercising and it (Steps2Health) definitely was encouraging. One thing (that was encouraging) was hitting your markers when you’ve made it there, and there was interesting information… Having that didn’t overwhelm me and I could make the markers they were setting up for me.

P22

A few participants noted that they appreciated that the Fitbit device gave them credit for steps when they were walking without their smartphones, and some stated that they wished the *Steps2Health* intervention would also be able to celebrate achievements in other forms of exercise that did not necessarily increase step count (eg, swimming).

#### Theme 2: Autonomous Motivation

##### Enjoyment of Journey Messages

Messages targeting *autonomous regulation* and *competence or self-efficacy* received the highest ratings in the follow-up survey (Figure 2). These messages comprised the bulk of the *Steps2Health* intervention. Participants generally found the intervention to be enjoyable and compelling. In total, 78% (29/37) of participants reported that they would recommend the intervention to a friend or family member (Figure 2). This met the a priori target of >75%, supporting the feasibility of the intervention*.*

In individual interviews, 14 of the 15 participants (93%) said that they would participate in another journey if offered. Some interviewees indicated that they were motivated to be more active to see what was next on the journey. One participant said:

It’s very easy in survivorship to have the world be overwhelming, you fall behind. You move slower than before, and it helped keep it a priority. This was very helpful—some of these messages were really pleasant, and I wanted to see what was going to come next.

P60

Another participant said:

I am coming from a year where fitness wasn’t my strong point after treatment but I enjoyed the messages and was sad that it ended…I was excited to read the messages, I talked about them with my kids and it was like a family event. I showed them the milestones about the bridges we crossed. It was really neat.

P29

However, a few participants said that the text messages were not very enjoyable or motivating to them. One said:

Text messages were annoying—they came at random so I blocked it.

P34

Despite this, the Fitbit feedback and app functions were almost universally well received.

##### Linked Media Content

The use of linked media content in the messages was almost categorically cited as being a good idea, but in individual interviews, many participants said that they did not often access the links. The primary reason given for this was that the participants were too busy to give these messages enough attention when they were received. Participants appreciated that the content would remain in their text message history, but many said that they seldom went back to access them. Recommendations from participants included providing a more readily accessible repository of this content and providing messages that were more individually relevant or personally tailored (eg, messages of prescriptions of physical activity that take into account the user’s general fitness level, personal preferences, and/or mobility limitations).

##### Motivation for Physical Activity

Although participants in general seem to have enjoyed the intervention content, results concerning whether participants felt the text messages effectively motivated them to engage in more physical activity were mixed. When asked in individual interviews, participants tended to respond that the messages did make them feel more motivated to exercise. However, qualitative data analysis suggested that participants often did not disentangle the effects of the *Steps2Health* intervention messages from those provided by the Fitbit device or app (eg, messaging from the Fitbit device that is provided if or when the user reaches 10,000 steps in a day). Furthermore, the results of the quantitative data analysis were mixed, with 51% (19/37) of the sample agreeing or strongly agreeing with the statement “Receiving the text messages motivated me to increase my physical activity,” whereas 22% (8/37) disagreed or strongly disagreed with this statement and 27% (10/37) indicated they were neutral (Figure 2).

##### Participant Engagement

Some intervention messages explicitly requested a response, and in this study, we conceptualized participant engagement as the response patterns to these intervention messages. Messages requesting a response targeted either *autonomy* or *relatedness*. The response rate for the former was 73% (45/62) and that for the latter was 36.7% (79/215). These results did not meet the a priori target of >80% and did not support intervention feasibility*.* In individual interviews, participants indicated that they were sometimes unaware that they had missed these texts. This may have been partly because of the fact that these text messages could sometimes become buried by subsequent messages if participants were particularly active or if they had not synchronized their devices in a while. Participants indicated that they were often on the move when these messages were received and were unable or unwilling to attend to the messages at that time. The response rate for messages pertaining to *relatedness* was particularly low, possibly owing to lower participant satisfaction with these messages, detailed below.

#### Theme 3: Relatedness

Participants’ satisfaction with the intervention messages targeting *relatedness* was mixed. Quantitative data indicated that these messages were less well received than the other types of messages, with 24% (9/37) of participants disagreeing or strongly disagreeing with the statement, “The text messages from Ruby were enjoyable to me” (the least well rated of the specific types of messages, as shown in Figure 2). In individual interviews, some participants were very positive about this aspect of the program:

I was waiting for those messages every day… I feel like [Ruby] was my coach.

P30

Some reported that they did not believe that this approach had much utility in fostering feelings of *relatedness*; one participant said:

Ruby, I just didn’t do well on that. I am not one of those people who does a lot of texting.

P68

Others said that they liked the idea of having a supportive role model but became disillusioned with this aspect of the intervention when they received only brief, automatic replies in response to the thoughtful messages they sent.

As noted earlier, the participant response rate to questions that explicitly requested a response was low (79/215, 36.7%). During interviews, participants offered several suggestions for improving this aspect of the intervention. One suggestion was to avoid using generic replies to participant responses to mimic an actual conversation. Another suggestion was to provide multiple character avatars for users to choose from, which might enhance relatability:

The idea of selecting among two or three (role models) would be awesome, and that may be all you need. At most four. We aren’t going to be chatting, so don’t try to make it feel like we are chatting.

P60

This point was emphasized by the male survivors we interviewed, who felt they did not have much in common with the female role model character. To foster feelings of *relatedness,* some participants suggested making use of text messaging or social media platforms to connect users to one another. One participant said:

If we could do a group text… that way maybe if someone was having a hard day we could encourage each other.

P4

However, this notion was met with mixed opinions. According to another participant:

My trust level in terms of responding to other people I do not know—I just don’t do that.

P68

### Intervention Effects

#### Autonomous Regulation

There was a small, statistically significant effect of time such that participants’ *autonomous regulation* scores tended to increase by 8% from before to after the intervention (B=0.16; 95% CI 0.01 to 0.30; *P=*.04; *d*=0.49). The results did not support a group-by-time interaction for *autonomous regulation* (*P=*.59).

#### Godin Leisure-Time Exercise Questionnaire

Linear mixed model results indicated a statistically significant group-by-time interaction for physical activity as measured by the Godin Leisure-Time Exercise Questionnaire (B=10.78; 95% CI 3.54 to 18.02; *P=*.005). Assignment to the experimental group was associated with increased self-reported physical activity behaviors (*d*=0.64). On average, participants in the experimental group had an increase of 52% in their Godin Leisure-Time Exercise Questionnaire score, whereas participants in the comparison group tended to have a slight decrease in their score (Multimedia Appendix 3).

#### Step Count

**Figure 3:**
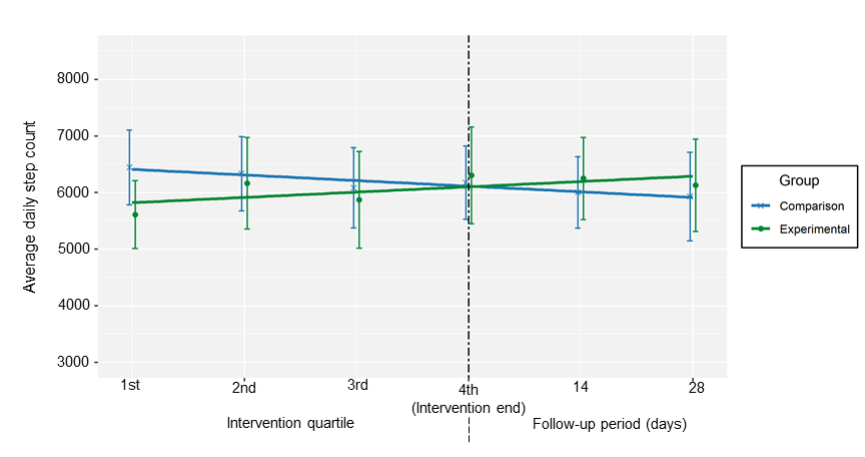
Mean device-measured daily step counts with linear trend lines by group over the study period. Quartiles are presented for the intervention period because the intervention duration differed for each pair. The median intervention period was 30 days (IQR 23-51 days).

The intraclass correlation coefficient for participants’ mean daily step count per journey quartile was 0.73 (95% CI 0.64 to 0.81). The linear growth model results indicated a statistically significant group-by-time interaction for step count during the intervention (Table 3; Multimedia Appendices 4-6). The experimental group participants were more likely to increase their step counts during the intervention (*d*=0.28) than the comparison group participants, and this trend extended into the follow-up period (Figure 3). The linear growth model suggested that the experimental group participants tended to have a lower step count earlier in the intervention than the comparison group participants, but this difference was not statistically significant.

**Table 3:** Parameter estimates for the linear growth model of the mean daily step count per intervention quartile as a function of group assignment.

Effect		Estimate SE	SE	*P* value	95% CI
**Fixed**					
	Intercept	6473.46	324.26	<.001	5837.91 to 7109.01
	Time	–107.41	96.06	.27	–295.69 to 80.87
	Group	–885.80	466.38	.06	–1799.90 to 28.30
	Days of valid wear	–287.14	227.86	.21	–733.75 to 159.47
	Group by time	322.08	136.77	.02	54.01 to 590.15
**Random at level 2 (between persons)^a^**					
	Intercept	2,648,712	628,223	<.001	1,417,395 to 3,880,029
	Slope for time	1284	21,090	.48	–40,052 to 42,620
**Random at level 1 (within person)^b^**					
	Residual	1,399,430	207,992	<.001	991,766 to 1,807,094

^a^The correlation between the intercept and slope for time was 0.90.

^b^The autocorrelation was 0.21.

## Discussion

### Principal Findings and Comparison With Prior Work

*Steps2Health* is a physical activity intervention that pairs game design elements with wearable mHealth technologies to target SDT constructs. In general, the results of our study support the feasibility of sending text messages for physical activity promotion contingent on real-time step counts to insufficiently active cancer survivors. One exception is that the participants’ response rate to the text messages requesting a response was low; this corroborates previous literature that has identified participant engagement as a challenge to mHealth interventions [41,42]. Our results also corroborate previous studies’ findings supporting the feasibility and acceptability of behavioral interventions centered on providing wearables to cancer survivors to promote their physical activity [43]. Over 96% (75/78) of the participants in this study completed the intervention, and similar to other studies, the percentage of valid wear time of the Fitbit device in this study sample was high [43,44]. Participants generally rated their satisfaction with the intervention as high but had mixed ratings for aspects of the intervention centered on targeting *relatedness*. Questionnaire data suggested that the *Steps2Health* intervention increased self-reported moderate-to-vigorous physical activity levels from pre- to postintervention. Device-measured data suggested that the *Steps2Health* intervention was associated with a greater increase in daily step count over the course of the study period; the comparison group appeared to have a higher step count early in the intervention period, which steadily declined, whereas the experimental group had a lower step count early on, which steadily increased.

The game design elements of the *Steps2Health* intervention are in line with the recommendation by Nicholson [45] that, to foster long-term behavior change, gamified intervention approaches should be centered on providing information that engenders meaningful connections. Beyond providing performance feedback, which may not always be relevant to insufficiently active cancer survivors [29], the *Steps2Health* intervention messages were designed to build *autonomous motivation* by fostering curiosity, unexpected adventures, and playfulness in the context of cancer survivorship. The results of this study support the general acceptability of this approach and the feasibility of using the approach in real time. Survey and qualitative data from individual interviews indicated that participants found the messages targeting *autonomous motivation* to be pleasant and compelling and that they would recommend the experience to friends and family. The messages targeting *autonomy* and *competence or self-efficacy* were also well rated.

Compared with the messages targeting *autonomy* and *competence or self-efficacy*, the messages targeting *relatedness* were not as well received, and the feasibility criterion associated with the response rate to these messages was not met. Enhancing *relatedness* has the potential to promote physical activity in cancer survivors; however, the marked heterogeneity of this population introduces challenges [29,46]. Some individuals may be more receptive to having this need fulfilled by constructive social support, whereas others may benefit more from healthy competition [47]. The incorporation of more granular, individualized tailoring may enable future mHealth interventions to meet this need for a greater diversity of individuals. In this study, participants recommended providing multiple role model characters to choose from, which they felt would help to enhance relatability. Anticipating our study sample, we created a female role model, but our findings support the notion emphasized in previous literature that participant *self-tailoring* may foster individual autonomy and connection to digital interventions (eg, allowing users to choose role models whose background and story most resonate with their own) [41]. Some participants suggested including aspects of social media as a part of the intervention experience; however, this idea was met with mixed opinions and some skepticism.

One increasingly viable intervention option that may serve to enhance *relatedness* in cancer survivors in mHealth interventions to some degree is the use of more sophisticated conversational technology, such as automated conversational agents, known as chatbots. Chatbots are increasingly being used as health intervention components [48,49] and this emerging technology could help increase participant engagement and/or facilitate feelings of *relatedness* for some individuals while preserving the scalability of the intervention. *Relatedness* is theorized to influence health-related outcomes both directly and by impacting *autonomous regulation* [13]. Accordingly, targeting *relatedness* should be a priority for interventionists but the degree to which this psychological need can be met via electronic means is unclear. More research is needed to determine how mHealth interventions can meaningfully impact this construct in cancer survivors.

Research indicates that mHealth programs generally have high rates of participant discontinuation [50] and that participant engagement plays an important role in influencing the decision to continue an intervention [41,42]. In this study, Fitbit device wear was relatively high, and participants tended to rate the intervention favorably. However, the messages that requested a response were not well received. Individual interviews revealed that one potential barrier to this aspect of participant engagement was that the messages requesting a response sometimes required participants to engage in deeper reflection. This was generally perceived as inappropriate for the context in which participants tended to receive the messages (ie, when they were more active). Thus, one lesson learned from this study is to carefully consider the context in which intervention messages are to be received. We found that participants were not very likely to review past text messages, and this sentiment was echoed by participants’ admission that they seldom accessed previously sent links to resources for healthy living. Future mHealth physical activity interventions could potentially increase engagement by configuring messages to be sent during lulls in physical activity that follow bouts of exercise (eg, at the conclusion of bouts of physical activity as registered by meeting a threshold level of Fitbit Active Minutes over a predetermined time period). This may be more conducive to participant engagement and serve as a useful opportunity to provide positive reinforcement and/or self-reflective, just-in-time support [51].

Our operationalization of participant response rate in this study was a summative measure of frequency. Future research should investigate what constitutes *effective engagement* or a broader conceptualization of engagement that relates more directly to desired intervention outcomes [41,42]. In this study, although the participant engagement feasibility criterion corresponding to the text message response rate was not met, participants in the experimental group tended to increase their physical activity levels more than their counterparts in the comparison group. A successful aspect of this study that helped facilitate participant engagement and high device wear was our inclusion of automated messages that were automatically sent if participants did not synchronize their Fitbit device for a prescribed period of time. The timestamp of users’ last synchronization is available through the Fitbit application programming interface. In this study, we were able to use these data to help increase participants’ engagement and data collection in a manner that minimized the use of study resources.

Although the experimental group reported increases in physical activity, the study results were mixed regarding whether participants felt that the *Steps2Health* messages motivated them to increase their physical activity levels. Interestingly, Zuckerman and Gal-Oz [52] noted a similar phenomenon regarding a discrepancy between the perceived and empirical effects of a gamified physical activity intervention. Participants may not necessarily attribute lifestyle changes to apparently effective gamification elements. Furthermore, we found no evidence that group assignment was associated with an increase in *autonomous regulation*. A review conducted by Johnson [53] revealed that the effects of gamification-centered wellness interventions are strongest for physical activity–related behavioral outcomes but weaker for motivation-related cognitions. Nonetheless, both the experimental and comparison groups exhibited a small, statistically significant increase in *autonomous regulation.* The provision of a wearable device alone could impact *autonomous regulation* in this population; however, this notion should be met with caution. The provision of consumer wearable technologies alone may undermine physical activity–related motivations in some populations [54] by emphasizing less autonomous forms of motivation, inducing anxiety and stigma, or by imposing unattainable or inappropriate norms [55].

Our findings indicated that the *Steps2Health* intervention was associated with an increase in self-reported exercise and device-measured step count during the intervention. Survey analyses provided evidence for a medium effect size from baseline to follow-up that is consistent with other mHealth-based behavioral interventions for promoting cancer survivors’ physical activity [56,57]. This finding was supplemented by an analysis of device-measured step counts over the intervention period. The comparison group participants’ step count was initially high but trended steadily downward. This is concordant with a waning *novelty effect* associated with physical activity device technologies [12]. The gamification aspects of the *Steps2Health* intervention may have influenced participants to gradually increase their step count. Future studies should further investigate these dynamics, as long-term physical activity maintenance is needed to best realize the diverse health benefits of physical activity. In some cases, it may be prudent to delay game design intervention components to capitalize on their effects and the *novelty effect* associated with the provision of physical activity tracking technologies. In fact, the Maintain IT Model of health behavior change posits that interventions centered on self-regulation may be appropriate for health behavior initiation, whereas interventions centered on precipitating a more centered and empowered state, consistent with SDT, may be more effective for leading to long-term health behavior maintenance [58].

### Study Limitations

The establishment of the acceptability and feasibility of digital behavior change interventions is critically important, given their high initial costs and high rate of discontinuation of use. Furthermore, the CONSORT (Consolidated Standards of Reporting Trials) 2010 Statement Extension to Randomized Pilot and Feasibility Trials outlines the need for prespecified assessments of pilot trial objectives [59]. Unfortunately, there is a lack of consensus on the established criteria to aid researchers in determining the feasibility and acceptability of digital behavior change interventions. In this study, we set an a priori criteria for assessing the feasibility of *Steps2Health* based on the intervention’s unique components (eg, text messaging contingent on physical activity tracker device–derived data), data observed in comparable studies (eg, for physical activity tracker wear), and the theoretical orientation of our intervention (ie, the emphasis on intrinsic motivation in SDT). However, we acknowledge that these criteria, and the thresholds we set for them, may not be applicable to other studies (or necessarily the ideal benchmarks for evaluating the feasibility of *Steps2Health*). Establishing universal quantitative criteria and corresponding thresholds may be a challenge, given the heterogeneous and rapidly evolving nature of digital behavior change interventions, but establishing basic metrics for commonly occurring physical activity intervention components may facilitate the conduct of rigorous research. Future research on this topic could make a meaningful contribution to the literature.

This study was limited by its small sample size and the use of convenience sampling recruitment methods. Participants may have been particularly motivated to change their physical activity levels from the outset, which may have interacted with the intervention components. The generalizability of the study’s findings is limited by the fact that the study sample was relatively well educated and mostly female. Another limitation of this study is that we did not audio record the in-depth interviews and used field notes instead. Loss of details from field notes may have increased the risk of bias in the qualitative analysis. Furthermore, although every effort was made to capture insightful quotations accurately, some errors may have been introduced in this process. Nevertheless, associated threats to validity were reduced by our relatively straightforward applied research questions, short interviews, study staff members exclusively dedicated to taking field notes, and a research protocol specifying that the interviewer separately provided field notes immediately after all interviews. The measurement of physical activity via self-report has known limitations, and this is also true for consumer-grade wearable devices. Fitbit devices tend to overestimate step counts in free-living conditions, particularly for individuals with chronic disease and/or mobility limitations [38]. However, this study’s limitations are offset by its notable strengths, including a randomized controlled design, a novel intervention approach, minimal loss to follow-up, the use of both quantitative and qualitative methods, and intervention qualities that lend themselves readily to widespread dissemination.

### Conclusions

The findings of this study support the feasibility and acceptability of using the gamification of real-time step counts to increase cancer survivors’ physical activity. Both the experimental and comparison groups increased their *autonomous regulation* for physical activity, and assignment to the experimental group was associated with increased physical activity. Coupling game design elements with wearable technologies is technically feasible and acceptable to cancer survivors and is potentially effective. More research is needed to develop these approaches as they have the potential to have a meaningful impact on public health.

## Appendix

Multimedia Appendix 1 Steps2Health interview guide.

Multimedia Appendix 2 Conceptual model for Steps2Health intervention.

Multimedia Appendix 3 Mean Godin Leisure-Time Physical Activity Questionnaire scores by group at baseline and follow-up. Error bars indicate 95% CIs.

Multimedia Appendix 4 Spaghetti plots of mean (thick) and subject-specific (thin) step count trajectories for the comparison and experimental groups during the intervention period.

Multimedia Appendix 5 Raw and fitted step count trajectories for individual participants in the experimental group.

Multimedia Appendix 6 Raw and fitted step count trajectories for individual participants in the comparison group.

## Abbreviations

BREQ-3: Behavioral Regulation for Exercise Questionnaire-3
mHealth: mobile health
MMS: multimedia messaging service
SDT: Self-Determination Theory
