# Supplementary material for: Gamified Text Messaging Contingent on Device-Measured Steps: Randomized Feasibility Study of a Physical Activity Intervention for Cancer Survivors
Source: JMIR Mhealth Uhealth. 2020 Nov 24;8(11):e18364. doi: 10.2196/18364 (PMC7723748; doi:10.2196/18364)
Supplement: Multimedia Appendix 1 [file mhealth_v8i11e18364_app1.docx]

**Interview Guide.**

1. Please tell me all about your experience with the messaging program *(use probes if necessary: how useful was the program? what problems did you have? etc.)*
2. What did you like most about the messaging program?
3. What did you like least about the messaging program?
4. If you could change something about the information in the messages, what would it be? Why?
5. Please tell us about your experience with the technological aspects of this messaging program.
   1. What worked for you, if anything? What difficulties did you encounter, if any?
   2. Did the way everything was presented make sense to you?
   3. How can we improve upon the technological aspect of this program?
6. Do you think the messaging program caused you to be more physically active? Why or why not?
   1. What specifically did/would you find useful?

*The messages received throughout the Steps2Health program are divided up into four categories. Use these example messages to describe each category as needed for the questions below.*

- ***Messages with journey destination content***
  - - *E.g., “We begin our island hopping adventure in Onomichi, on the biggest island in Japan. At the center of this city is a park with a historic Japanese temple, Senkō-ji.”*
- ***Messages associated with the two optional mini-journeys*** *(to the “Town of Hills and Cats” and “Fluffy Rabbit Island”).*
  - - *E.g., “You have arrived at Okunoshima Island. Hundreds of bunny rabbits roam freely on this peaceful little island.”*
- ***Supportive messages from Ruby*** *(the person who had been on the journey before)*
  - - *E.g., “At the Ōyamazumi Shrine, I reflected on how far I’ve come, how I’ve have grown in so many ways. I’d invite you to do the same. What are you most grateful for?”*
- ***Messages aiming to help build confidence for increasing physical activity***
  - - *E.g., “Maintaining a healthy lifestyle after treatment is possible. Stay focused on your goals and keep up your healthy attitude.”*

1. Please tell me about your experience regarding the **messages with journey destination content.**
   1. Were there any journey checkpoints that you particularly liked? Disliked? Why?
   2. What was your experience with the linked media content? About what percentage of the links do you think you accessed?
      1. What content was most useful or enjoyable (list the linked media content in messages if necessary)? Why?
      2. What was least useful or enjoyable? Why?
      3. What additional content do you think we should add, if anything?
2. Please tell me about your experience regarding the **messages associated with the two optional mini-journeys/excursions.**
   1. Did you opt in or out of them? Why?
   2. Were they fun or useful for you? Why or why not?
3. Please tell us about your experience regarding the **supportive messages from Ruby.**
   1. What about these messages was fun or useful for you, if anything? What didn’t work?
   2. What suggestions do you have for sending messages that might help to make participants feel more supported?
4. Please tell us about your experience regarding the **messages aiming to help build confidence for increasing physical activity** (provide examples)**.**
   1. What about these messages was fun or useful for you, if anything? What didn’t work?
   2. What suggestions do you have for sending messages that might help to make participants feel more confident that they can be more physically active?
5. What else do we need to know about this program? What have I not asked about that you think we should know?
6. If we had another journey developed right now, would you want to participate in it?
7. If we developed another journey, where would you want to go?
8. What made you participate in this study?
   1. What did you expect?
9. Is there anything else that we didn’t ask about that you think we should know?
