# Supplementary figures and images for: Gamified Text Messaging Contingent on Device-Measured Steps: Randomized Feasibility Study of a Physical Activity Intervention for Cancer Survivors
Source: JMIR Mhealth Uhealth. 2020 Nov 24;8(11):e18364. doi: 10.2196/18364 (PMC7723748; doi:10.2196/18364)

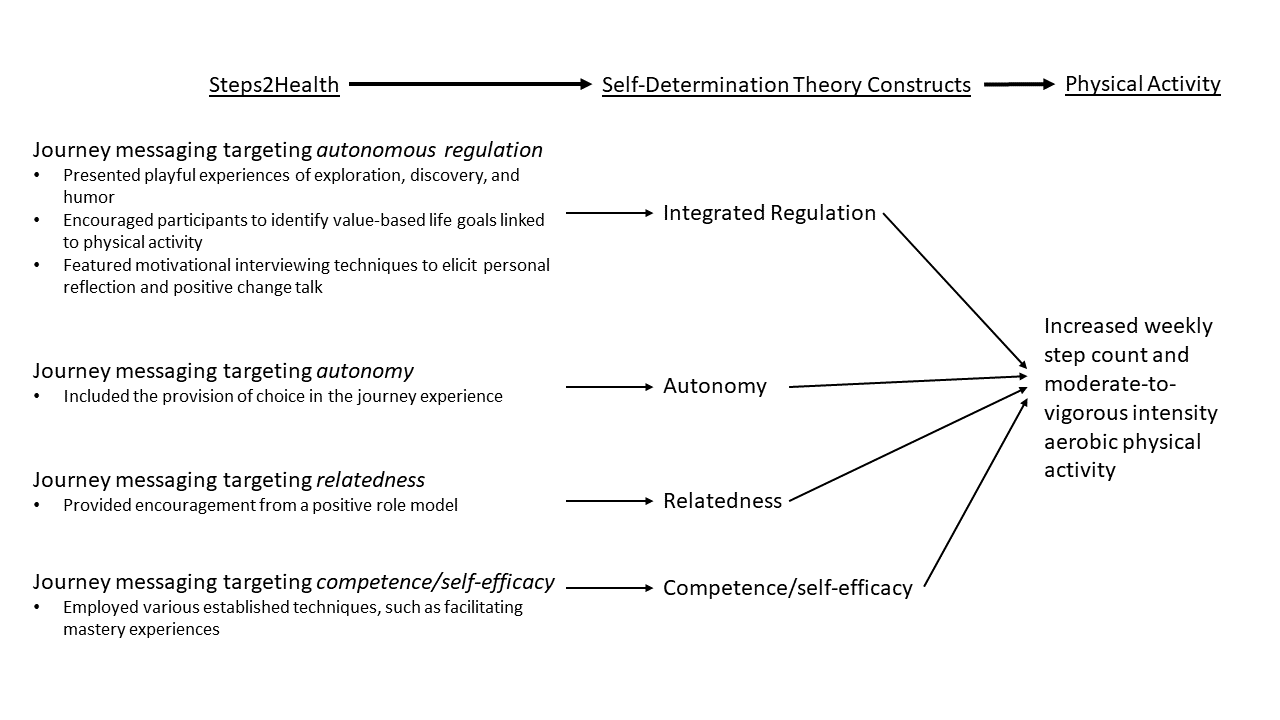

Supplement: Multimedia Appendix 2 [file mhealth_v8i11e18364_app2.png]

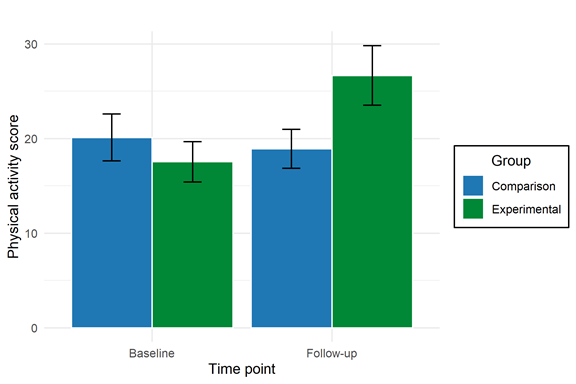

Supplement: Multimedia Appendix 3 [file mhealth_v8i11e18364_app3.png]

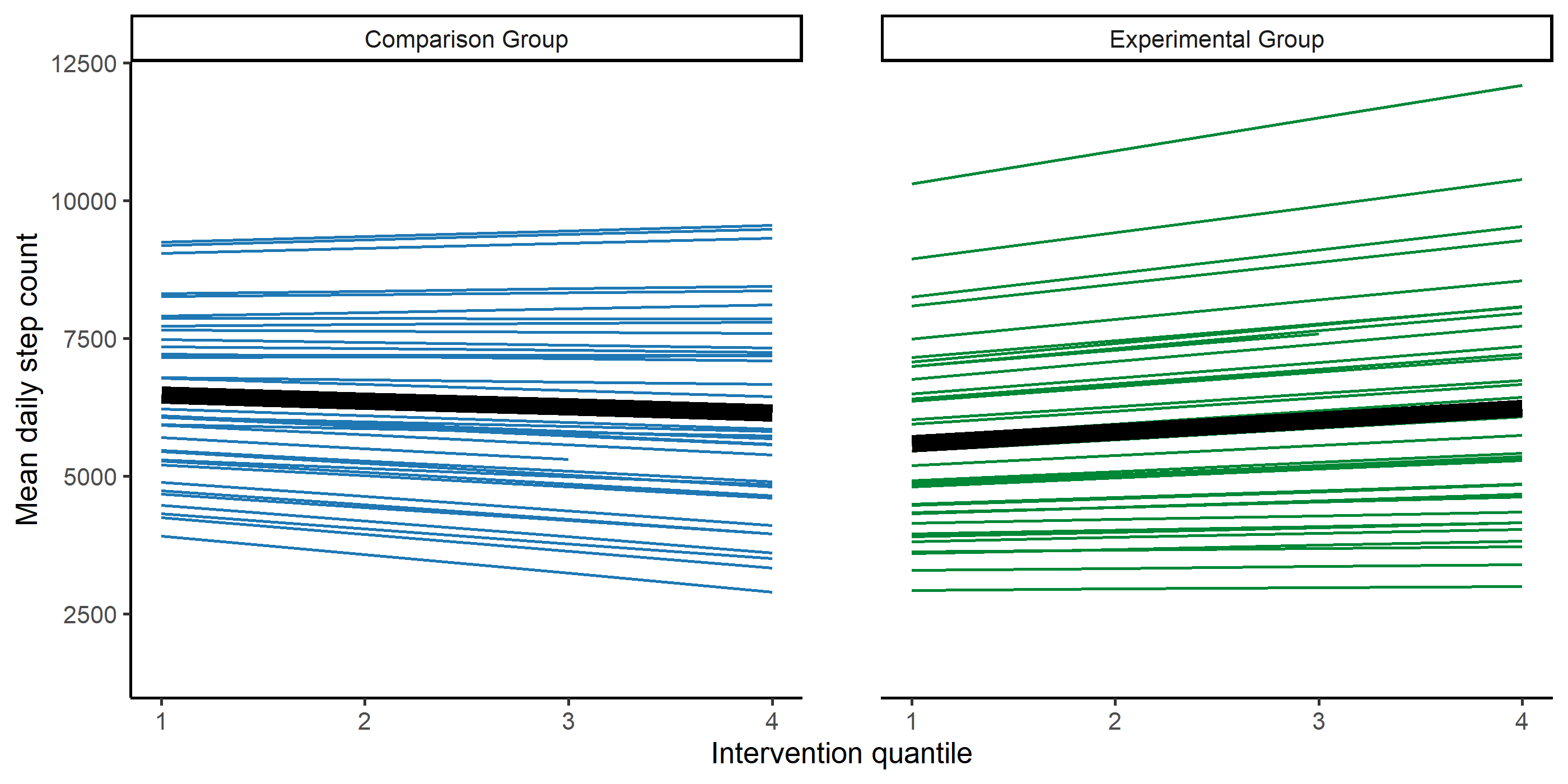

Supplement: Multimedia Appendix 4 [file mhealth_v8i11e18364_app4.png]

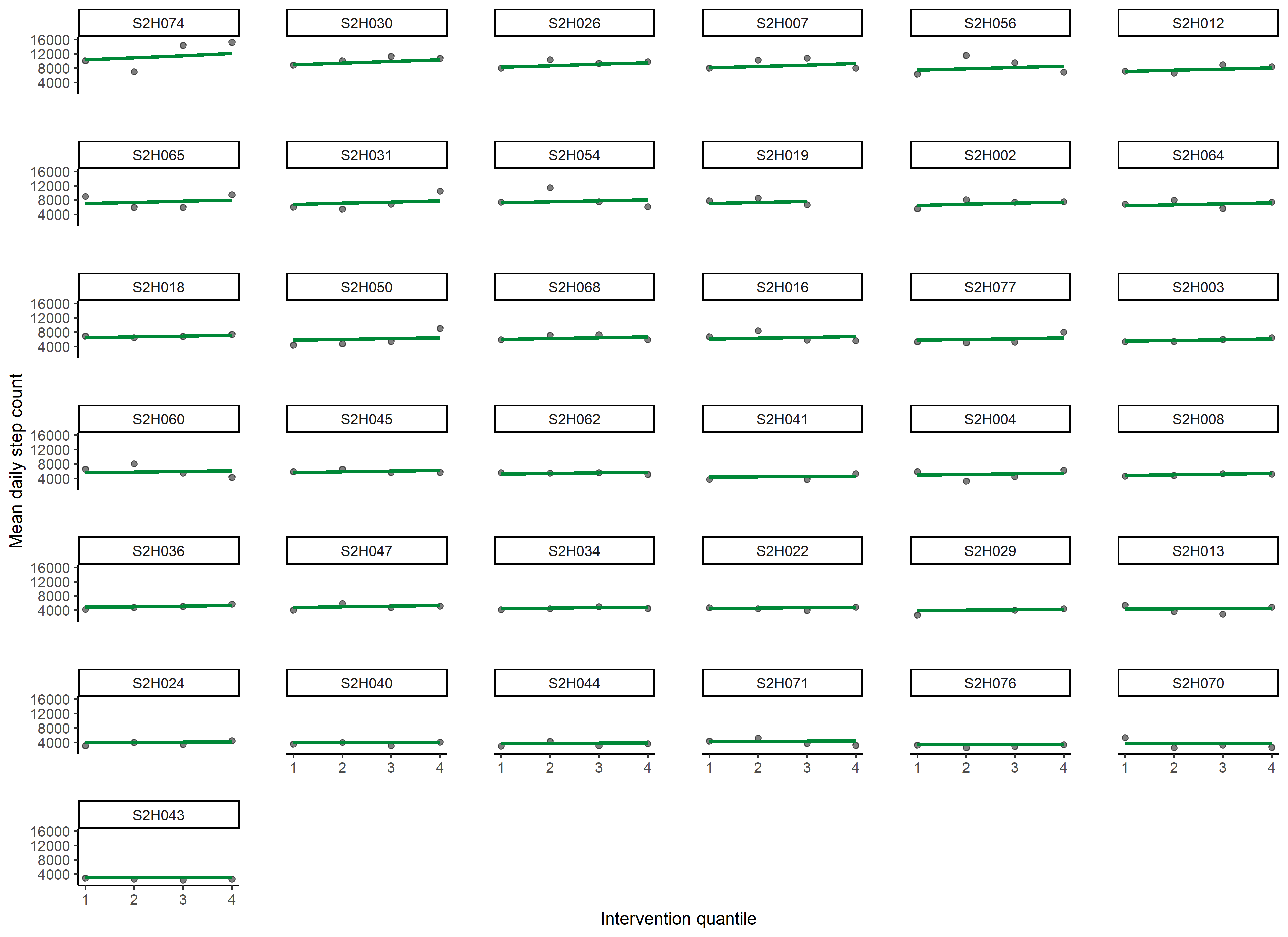

Supplement: Multimedia Appendix 5 [file mhealth_v8i11e18364_app5.png]

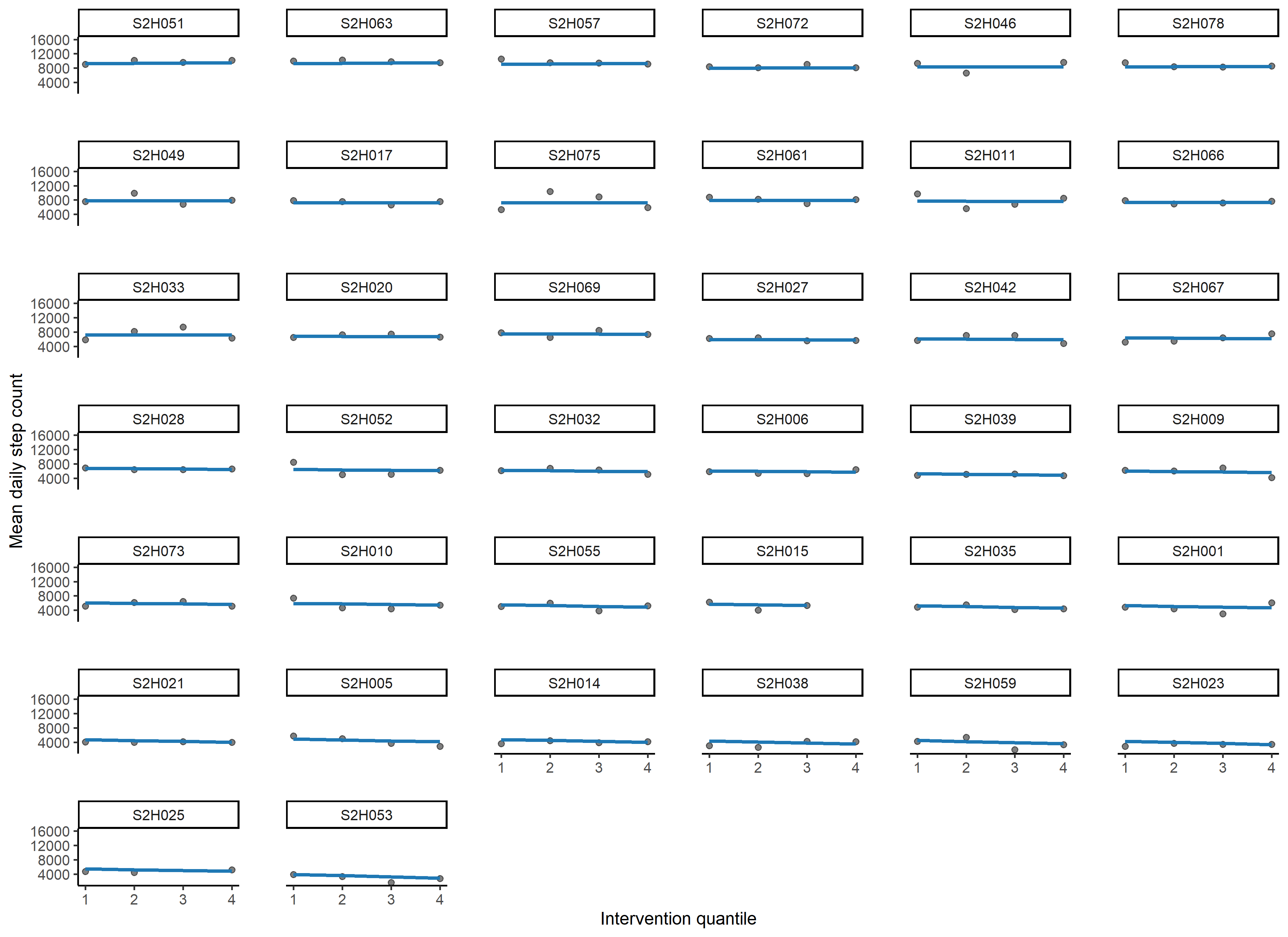

Supplement: Multimedia Appendix 6 [file mhealth_v8i11e18364_app6.png]
